# Identification of a 5-lncRNA-Based Signature for Immune Characteristics and Prognosis of Lung Squamous Cell Carcinoma and Verification of the Function of lncRNA SPATA41

**DOI:** 10.3389/fgene.2022.905353

**Published:** 2022-08-29

**Authors:** Sheng Huan, Miao Chen, Sumin Sun, Yanling Zhong, Yu Chen, Yihao Ji, Guoping Yin

**Affiliations:** ^1^ Nanjing Hospital Affiliated to Nanjing University of Chinese Medicine, Nanjing, China; ^2^ Department of Anesthesiology, Nanjing Second Hospital, Nanjing, China; ^3^ Department of Surgery, Nanjing Second Hospital, Nanjing, China; ^4^ Nanjing University of Chinese Medicine, Nanjing, China; ^5^ Nanjing Medical University, Nanjing, China; ^6^ Department of Critical Medicine, Nanjing Second Hospital, Nanjing, China

**Keywords:** lncRNA, lung squamous cell carcinoma, nomogram, prognosis, SPATA41

## Abstract

Lung squamous cell carcinoma (LUSC) is one of the most lethal cancers worldwide. Traditional tumor-node-metastasis (TNM) staging system has many insufficiencies in predicting immune characteristics, overall survival (OS), and prognosis of LUSC. LncRNA is currently found involved in tumor development and effectively predicts tumor prognosis. We screened potential tumor-related lncRNAs for immune characteristics and constructed a nomogram combining lncRNA and traditional clinical indicators for prognosis prediction. We obtained the large-scale gene expression profiles of samples from 492 LUSC patients in The Cancer Genome Atlas database. SPATA41, AL034550.2, AP003721.2, AC106786.1, and AC078889.1 were finally screened to construct a 5-lncRNA-based signature. The risk score of the signature divided patients into subgroups of high-risk and low-risk with significant differences in OS. Their area under the curve (AUC) reached more than 0.70 in 1, 3, and 5 years. In addition, compared with the high-risk subgroup, the low-risk subgroup exhibited a remarkably favorable prognosis and TME score, along with a higher immune infiltration score and lower TIDE score. The signature also significantly related to chemotherapy response, especially in cisplatin, vinorelbine, and paclitaxel. Importantly, the nomogram we constructed had good reliability with the assessment of the calibration chart and consistency index (c-index). GO and KEGG enrichment analysis indicated that co-expression mRNAs of the 5 lncRNAs were mainly focused on RNA splicing, DNA replication, and protein serine/threonine kinase activity. Functional assays demonstrated that SPATA41, one of the five OS-related lncRNAs, regulated invasion, migration, proliferation, and programmed death *in vitro*. In summary, our 5-lncRNA-based signature has a good performance in predicting immune characteristics and prognosis of LUSC patients.

## Background

Lung cancer is the most common chest tumor in the world, with a high incidence and mortality. More than 2.2 million people in the world have been newly diagnosed with lung cancer in 2021, accounting for 11.1% of all newly diagnosed cancer (Bu et al., 2020; Xu et al., 2020). Lung squamous cell carcinoma (LUSC), deriving from chronic stimulation and injury of bronchial columnar epithelial cells, takes up a large percentage of lung cancer (Sánchez Danés and Blanpain, 2018). Although LUSC grows slowly and metastases late, it is less sensitive to radiotherapy than undifferentiated lung carcinoma. In addition, LUSC lacks effective chemotherapeutic drugs compared with lung adenocarcinoma (LUAD) and has a poor prognosis. Presently, the best treatment for LUSC is still surgical resection, which has been demonstrated to have a good 5-year overall survival (OS) rate (Bozinovski et al., 2016; Socinski et al., 2018). Therefore, studies related to LUSC immune checkpoint blockade (ICB) therapy were urgent to perform for addressing the gap in this research field.

Tumor-node-metastasis (TNM) staging was often used to evaluate tumor development and prognosis in the past. However, many clinicians found it was not accurate enough in diagnosing LUSC due to complex disease pathology and high heterogeneity between patients (Lian et al., 2020). Recent research showed that lncRNA could regulate gene expression by influencing mRNA transcription, binding nucleic acids, and participating in posttranscriptional modification, such as DNA methylation and acetylation (Quinn et al., 2014; Engreitz et al., 2016; Storti et al., 2020). LncRNAs had been found to have great importance in the diagnosis, treatment, and prognosis of LUSC.

Tumor microenvironment (TME) plays a critical role in neoplasia and tumor development. Immune cells and immune stromal cells are the core components of TME, regulating tumor differentiation, proliferation, and metastasis. For example, a continuous abundance of specific T cell subtypes in tumors contributed to a better prognosis of patients (Jiang et al., 2018). Macrophage polarization was of great importance in subverting adaptive immunity and inducing tumor metastasis (Mantovani et al., 2002). Identifying the useful lncRNAs that influence the immune cells and immune stromal cells contributes to deciphering the carcinogenic mechanism of lncRNA.

Although previous studies have predicted and identified some molecular biomarkers in LUSC patients, most of them may have limited research meaning due to small sample sizes, differences in platforms, or a lack of combining diverse variables (Friedlaender et al., 2019; Li and Guo, 2020; Ma et al., 2020; Ren et al., 2020). For these reasons, we conducted this study to integrate relevant data and identify credible prognostic biomarkers for clinical guidance.

## Materials and Methods

### Workflow and Dataset Processing

**FIGURE 1:**
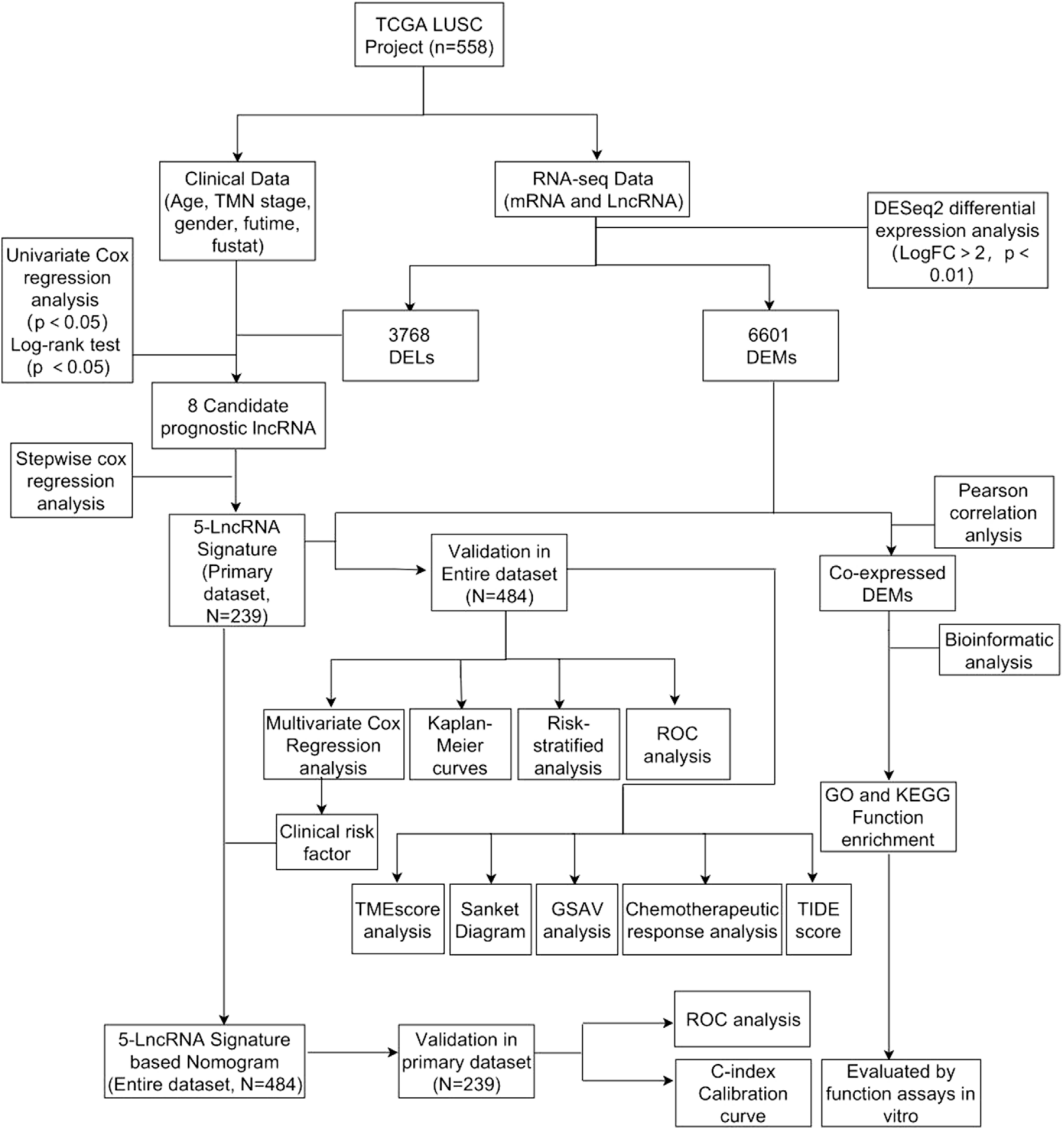
Flowchart of this study.

The flowchart was shown in Figure 1. We downloaded lncRNA expression data, mRNA expression data, and corresponding clinical data of LUSC from The Cancer Genome Atlas (TCGA, HYPERLINK “http://cancergenome.nih.gov/”) (Kourou et al., 2015; Gibson et al., 2016; Rokavec et al., 2017).

### Screening of Differentially Expressed lncRNAs and mRNAs

Differentially expressed lncRNAs (DELs) and differentially expressed mRNAs (DEMs) between LUSC samples and control samples were analyzed with the “DESeq2” package in R software. Then, we used “ggplot2” and “pheatmap” packages to draw volcanic and thermal maps of them (Robinson et al., 2010).

### Identification of lncRNA Signature and Calculation of Risk score

We constructed primary dataset (*n* = 484) and entire dataset (*n* = 239) to conduct data processing. Specifically, we first used univariate Cox proportional hazard regression (CPHR) to screen the lncRNAs significantly associated with the OS of LUSC patients, making them candidates (*p* = 0.01) (Tibshirani, 1997). Then we continued with multivariate CPHR analysis (stepwise model) to find the most suitable OS-related lncRNAs from the aforementioned candidate lncRNAs. Akaike information criterion (AIC) was used in order to avoid overfitting. We used “pheatmap” and “survminer” packages to draw thermal maps and survival probability curves.

### Sankey Diagram

We drew a Sankey diagram based on life-status, gender, TNM staging, and 5-lncRNA risk score with the "ggalluvial” package (Grazziotin et al., 2022).

### Nomogram and ROC Curves for Predicting OS

We drew ROC curves for predicting OS in the primary dataset and the entire dataset with the “survivalROC” package (Zhou et al., 2020).

### Generation of ImmuneScore, StromalScore, and ESTIMATEScore

The ESTIMATE algorithm was used to estimate the ratio of the immune-stromal components in TME for each sample with the “ggpubr” and “estimate” packages, exhibited in the form of three kinds of scores: Immune Score, StromalScore, and ESTIMATEScore, which positively correlated with the ratio of immune, stromal, and the sum of both, respectively (Yoshihara et al., 2013).

### Nomogram Construction and Reliability Assessment

We used the “RMS” package to draw the nomogram of the primary dataset and the entire dataset (Zhang and Kattan, 2017). The calibration chart and c-index were conducted to evaluate their value in calibrating and identifying the OS of LUSC.

### GSVA and Chemotherapeutic Response Prediction

We performed gene set variation analysis (GSVA) to evaluate the related immune cell and molecular pathway variation between the high-risk and low-risk groups with the “GSVA” package (Subramanian et al., 2005). Then, we conducted chemotherapeutic response prediction for LUSC based on our expression profiles with the “pRRophetic” and “ggExtra” packages. Five commonly used drugs were selected, namely, docetaxel, vinorelbine, cisplatin, paclitaxel, and gemcitabine (Reinhold et al., 2012). Moreover, tumor immune dysfunction and exclusion (TIDE) was also applied to predict the clinical response to immunotherapy of LUSC patients with the “ggpubr” package (Jiang et al., 2018).

### Enrichment Analysis of Co-related mRNA of the 5-lncRNA Signature

We used the “limma” package to select the mRNA co-expressed with these 5-lncRNA signatures (Pearson correlation coefficient >0.25). Then we used the “enrichplot” package to perform Gene ontology (GO) functional annotation and Kyoto Encyclopedia of Gene and Genomes (KEGG) functional annotation to verify the biological process and potential pathway (Yu et al., 2012). We also used the GSVA enrichment analysis to investigate the underlying biological activities between the high-risk subgroups and low-risk subgroups (Subramanian et al., 2005).

### Box Plots and Differential Gene Comparison

The microarray data of normal tissue and tumor tissue were downloaded from the GEO database (GSE73402) (http://www.ncbi.nih1.gov/geo). The raw data were downloaded as MINiML files. Box plots are drawn with the package “boxplot” (Zhou et al., 2020). Differential gene comparison was performed with Kruskal–Wallis rank sum test.

### Cell Culture and siRNA Transfection

BEAS-2B and H520 cells were cultured in RPMI-1640 medium (GIBCO, Shanghai, China), and SK-MES-1 cells were cultured in MEM medium (GIBCO, Shanghai, China). All media were supplemented with 10% fetal bovine serum (FBS) (GIBCO, Shanghai, China) and 1% penicillin and streptomycin (Solarbio, Beijing, China). The plasmids of SPATA41 siRNA were designed by Kaiji Biological Company (Nanjing, China) and transfected into H520 and SK-MES-1 cells with Lipofectamine 8000 according to the manufacturer’s instructions.

### RNA Extraction and Quantitative Real-Time PCR

The cells were lysed by Trizol. Total RNA was collected with isopropanol from Aladdin (Shanghai, China) and RNA content was determined by an enzyme labeling instrument. Total RNA was reverse-transcribed into cDNA with Hifair^®^ II 1st Strand cDNA Synthesis Kit from Yeasen (Shanghai, China). The cDNA was amplified with Hieff Q real-time PCR SYBR Green main mixture from Yeasen (Shanghai, China) and detected through Applied Biosystems 7500 from Thermo Fisher (Massachusetts, USA) according to the manufacturer’s instructions. The expression of the target gene was normalized to GADPH expression.

### Transwell Assay

The intervened cells (1 × 10^5^ cells/well) were seeded into the upper chamber of the transwell culture plate while 500 ul medium with 20% FBS was filled with the bottom chamber. After incubation for 24 h at 37 °C in 5% CO_2_, the cells were fixed in cooled methanol for 30 min and were stained with hematoxylin for 1 min, and washed with 1 X PBS. The representative images were obtained under an inverted light microscope.

### Wound-Healing Assay

The intervened cells (3 × 10^5^ cells/well) were seeded into a 6-well microplate until forming a fusion monolayer. We used the 200 μL sterile pipettes to make an artificial and uniform wound and carefully cleaned the unattached cells with 1 x PBS. Then the cells were incubated in a serum-free medium at 37 °C in 5% CO_2_. The representative images of 0 and 24 h were obtained under an inverted light microscope.

### Western Blotting

The total protein concentration was determined using the BCA protein analysis kit (Pierce, #23225). The same amount of proteins was added and separated by SDS-PAGE. Then it was transferred to PVDF membrane (microporous, IPVH00010) and blocked with 5% BSA for 1 h. The PVDF membranes were incubated with the primary antibody overnight and the secondary antibody for 2 h. Finally, the PVDF membranes were incubated with ECL (Pierce, #32109) and the strip strength was quantified by ImageJ software.

### Colony Formation Assay

The cells (1 × 10^5^ cells) were seeded into the culture chamber and the culture medium was covered. After incubation for 24 h at 37°C in 5% CO_2_, the cells were transfected with siRNA and then cultured for 7 days. Cell colonies suspended gradually formed in the medium on the matrix gel. The images were obtained with a common camera.

### Cell Counting Kit-8 Assay

The cells (5 × 10^4^ cells/well) were seeded into 96-well microplates and incubated for 24 h at 37°C in 5% CO_2_. After the intervention, cells were treated with 10 μL CCK8 reagents and incubated at 37°C for 4 h. The optical density was determined at 450 nm in a plate reader from Thermo Fisher (Massachusetts, USA).

### Annexin V-FITC/PI Double Staining Assay

The intervened cells were lysed, collected, and dissolved in buffer at the density of 1.0 × 10^5^ cells/mL. 100 μL sample solution was added with 5 μL FITC-conjugated Annexin V reagent and 5 μL Propidium iodide (PI) reagent and incubated for 15 min in a dark greenhouse. Percentages of cells within each cell death compartment (Q1, Q3, Q3, and Q4) were determined by flow cytometry. The results were analyzed by FlowJo software.

### Statistical Analysis

Our data were analyzed with the deviation of mean and standard. Results related to mapping were analyzed with GraphPad Prism 7.0. Statistical significance was determined using an unpaired Student’s t test for comparisons between two groups, while one-way ANOVA followed Tukey’s post hoc test was used for comparisons among more than two groups. The difference was considered significant when the *p* value was less than 0.05. Each experiment was repeated at least three times.

## Results

### Construction and Assessment of a 5-lncRNA Signature in Predicting Prognosis of LUSC Patients

In total, 510 LUSC tissue samples and 48 normal tissue samples were eventually included. After analyzing their lncRNA and mRNA expression profiles, we found that there were 6601 DEMs and 3768 DELs. Their volcanic and thermal maps were shown as Supplementary Figures S1, S2. Then, we combined clinical data with lncRNA expression and received data of 505 LUSC patients. After excluding 1 patient without survival time and 11 patients with insufficient survival data, relevant data of 492 patients were retained. 8 lncRNA were found to have a significant correlation with OS in LUSC patients (*p* < 0.05) (Supplementary Table S1). Kaplan–Meier curve was performed in accordance with the result of the univariate CPHR analysis (Supplementary Figure S3).

In order to further find the most suitable OS-related lncRNA, we continued to conduct multivariate CPHR. The result showed that five lncRNAs (SPATA41, AL034550.2, AP003721.2, AC106786.1, and AC078889.1) had the lowest AIC value and highest likelihood ratios (Table 1). The formula was shown as follows: Risk Score = (0.535 × Expression SPATA41) + (0.338 × Expression AC106786.1) + (−0.857 × Expression AL034550.2) + (−0.618 × Expression AP003721.2) + (−0.692 × Expression AC078889.1).

**TABLE 1:** 5 lncRNAs significantly associated with the OS of 492 LUSC patients.

Gene Name	Coefficient	Type	Down/up-Regulated	HR	95%CI	P Value
SPATA41	0.535264763	Risky	Up	1.707900371	1.239-2.354	0.001080441
AC106786.1	0.337590814	Risky	Up	1.401566884	1.067-1.841	0.015266432
AL034550.2	−0.857359527	Protect	Down	0.424280907	0.231-0.778	0.005590659
AP003721.2	−0.617768427	Protect	Down	0.53914624	0.353-0.822	0.004127988
AC078889.1	−0.692738229	Protect	Down	0.500204517	0.334-0.749	0.000781794

**FIGURE 2:**
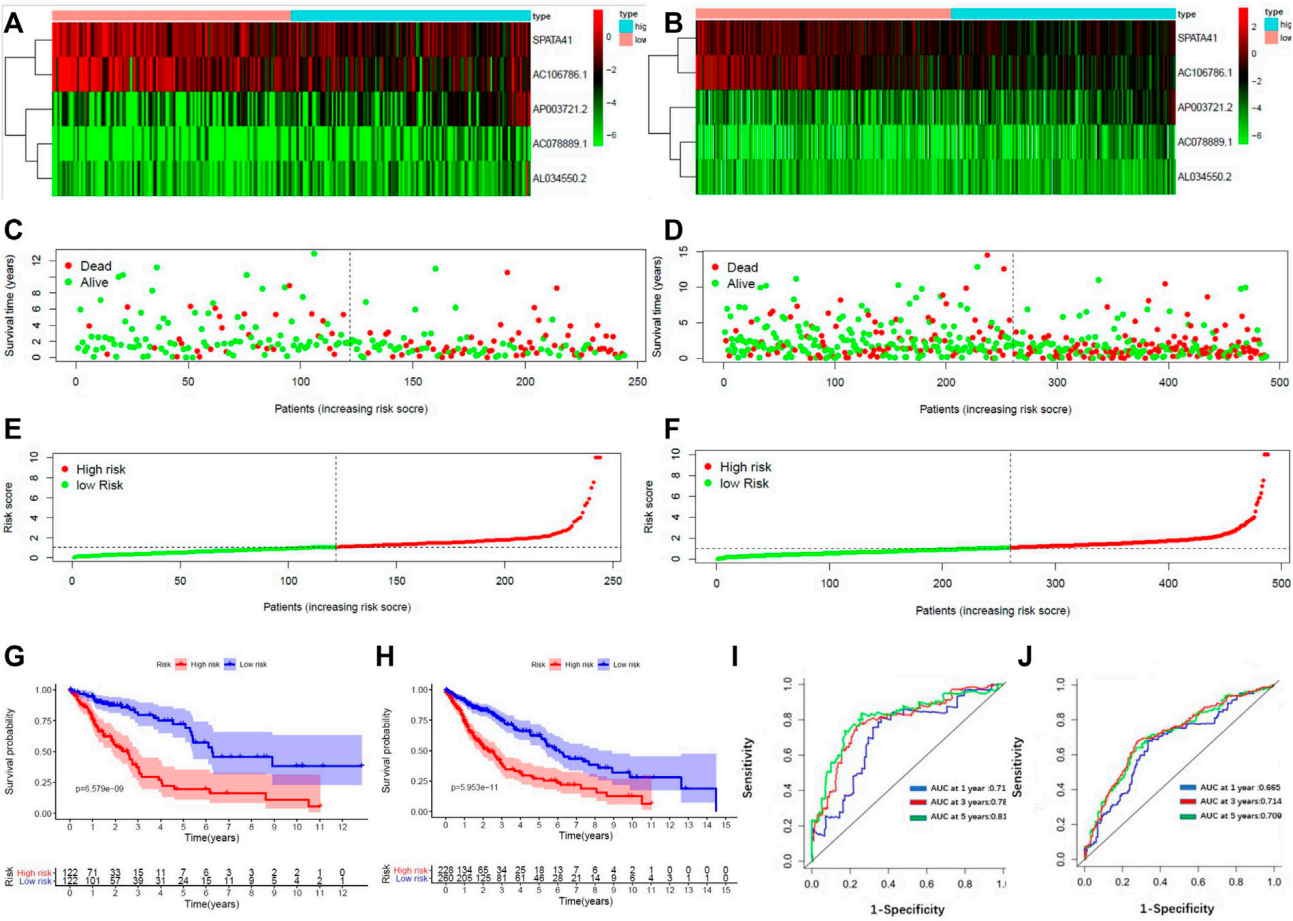
Construction of a 5-lncRNA signature in predicting prognosis of LUSC patients in the primary dataset and entire dataset. **(A)** The heatmap of 5-lncRNA signature in LUSC patients of the primary dataset. **(B)** The OS status of 5-lncRNA signature in LUSC patients of the primary dataset. **(C)** The risks score distribution of 5-lncRNA signature in LUSC patients of primary dataset. **(D)** The heatmap of 5-lncRNA signature in LUSC patients of the entire dataset. **(E)** The OS status of 5-lncRNA signature in LUSC patients of the entire dataset. **(F)** The risks score distribution of 5-lncRNA signature in LUSC patients of the entire dataset. **(G)** Kaplan–Meier curves are based on the 5-lncRNA signature of LUSC patients in the primary dataset. **(H)** Kaplan–Meier curves are based on the 5-lncRNA signature of LUSC patients in the entire dataset. **(I)** Time-dependent ROC curve based on 5-lncRNA signature of LUSC patients in the primary dataset. **(J)** Time-dependent ROC curve based on 5-lncRNA signature of LUSC patients in the entire dataset.

8 patients without TNM staging or age data were excluded. Within the remaining 484 LUSC patients, 239 patients were randomly classified into the subgroup of “primary dataset” and all 484 patients was assigned to the subgroup of “entire dataset”. The characteristics of these 484 patients in the entire dataset and 239 patients in the primary dataset are shown in Table 2. The five lncRNA expression profile, OS status, and risk score distribution of the primary dataset and the entire dataset were presented in Figures 2A–F. Patients were equally divided into a subgroup of high-risk and low-risk in accordance with median risk score. Kaplan–Meier curve also showed that patients in a subgroup of high-risk had a worse prognosis than that of low-risk in both the entire data set (*p* = 5.935e-11) and the primary dataset (*p* = 6.5790e-09) (Figures 2G, H).

**TABLE 2:** Baseline clinical characteristics of LUSC patients involved in this study.

Characteristic	Primary Dataset	Entire Dataset	*p* value
	*n* = 239	*n* = 484	
Age (years)	—	—	0.917534
≥65	157 (65.69%)	317(65.50%)	—
<65	82 (34.31%)	167 (34.50%)	—
Gender	—	—	0.642574
Female	59 (24.69%)	127 (26.24%)	—
Male	180 (75.31%)	357 (73.76%)	—
TNM stage	—	—	0.987752
I	115 (48.12%)	238 (49.17%)	—
II	82 (34.31%)	157 (32.44%)	—
III	39 (16.32%)	82 (16.94%)	—
IV	3 (1.26%)	7 (1.45%)	—
Tumor stage	—	—	0.719912
T0-T2	197 (82.43%)	393 (81.20%)	—
T3-T4	42 (17.57%)	91 (18.80%)	—
Lymph node metastasis	—	—	0.944372
Nx	2 (0.84%)	5 (1.03%)	—
No	154 (64.44%)	309 (63.84%)	—
Yes	83 (34.73%)	170 (35.12%)	—
Distant metastasis	—	—	0.830401
Mx	37 (15.48%)	77 (15.91%)	—
No	199 (83.26%)	400 (82.64%)	—
Yes	3 (1.26%)	7 (1.45%)	—

We constructed a receiver operating characteristic (ROC) curve based on the primary dataset and the entire dataset. As shown in Figures 2I, J, the area under the curve (AUC) of the 5-lncRNA signature reached 0.714 at 1 year, 0.789 at 3 years, and 0.810 at 5 years in the primary dataset while 0.665 at 1 year, 0.703 at 3 years, and 0.709 at 5 years in the entire dataset. The result of univariate and multivariate CPHR analysis in the entire dataset showed that the HR of the high-risk subgroup versus low-risk subgroup was 1.142 (*p* < 0.001, 95% CI = 1.082-1.205) and 1.152 (*p* < 0.001, 95% CI = 1.094-1.213), indicating that 5-lncRNA signature was independent of traditional clinical risk factors in predicting the prognosis of LUSC patients (Table 3). The univariate and multivariate CPHR analysis in the primary dataset showed consistent results (Supplementary Table S2). Risk stratification analysis was also performed on the entire dataset in consideration of the big sample of LUSC patients. We conducted a hierarchical analysis based on gender (female or male), age (≥65 or<65), TNM staging (I, II, III, or IV), T staging (T1, T2, T3, and T4), N staging (N0, N1, and N2), and M staging (M0 or M1). Each group was further assigned into subgroups of high-risk group and low-risk group in accordance with the risk score. As shown in the Kaplan–Meier curve, except for the condition of TNM III, TNM IV, T1, T4, N2, and M1, patients in the other conditions had a worse prognosis with higher risk score (Supplementary Figure S4).

**TABLE 3:** Univariate and multivariate Cox proportional hazards regression analysis of 5-lncRNA signature and clinical risk factors in the entire dataset.

Characteristic	Univariate analysis		Multivariate analysis	
	HR (95%CI)	*p*-Value	HR (95%CI)	*p*-Value
Age (≥65 *vs*. <65)	1.415 (0.996-2.010)	0.053	1.547 (1.079-2.219)	0.018
Gender (male *vs.* female)	1.256 (0.858-1.838)	0.241	1.271 (0.865-1.867)	0.222
TNM stage (III-IV *vs*. I-II)	1.248 (1.035-1.506)	0.020	1.128 (0.821-1.550)	0.458
Tumor stage (T3-T4 *vs*. T0-T2)	1.268 (1.033-1.557)	0.023	1.268 (0.932-1.600)	0.148
Lymph node metastasis (yes *vs.* no)	1.432 (0.895-2.291)	0.134	1.344 (0.727-2.486)	0.345
Distant metastasis (yes *vs.* no)	1.837 (0.583-5.784)	0.299	1.161 (0.307-4.387)	0.826
Risk score (high *vs.* low)	1.142 (1.082-1.205)	< 0.001	1.152 (1.094-1.213)	< 0.001

### Assessment of the TME Scores, Immune Characteristics, and Drug Sensitivity Related to the 5-lncRNA-Based Signature.

**FIGURE 3:**
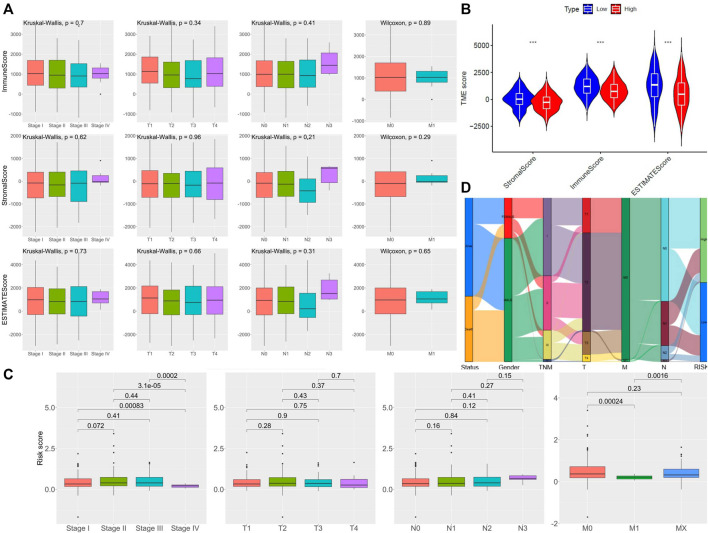
Assessment of the TME Scores among TNM staging and 5-lncRNA-based signature. **(A)** Comparison of ImmuneScore and StromalScore in different clinicopathological staging characteristics. **(B)** Comparison of risk score of LUSC patients of different TNM staging, T staging, N staging, and M staging. **(C)** Comparison of 5 lncRNA-based riskscore between LUSC patients of different TNM staging. **(D)** Alluvial diagram of subgroups based on 5-lncRNA signature with different life status, gender, and TNM staging.

For determining the relationship between the TME Scores with traditional TNM staging and 5-lncRNA-based signature, we analyzed the corresponding clinicopathological information. As shown in Figure 3A, there is no significant difference in TME Scores among TNM staging, T staging, M staging, and N staging. In contrast, the low-risk subgroup showed higher StromalScore, ImmuneScore, and ESTIMATEScore in comparison with the high-risk subgroup divided by 5-lncRNA-based signature (Figure 3B). However, there seemed to be no significant difference in 5-lncRNA based risk score among patients with different TNM staging, T staging, M staging, and N staging (Figure 3C). Sankey diagram was used to visualize the relationship between life status, gender, TNM staging, and risk score. The results showed that male patients had a more terrible life status and patients in the high-risk subgroup tended to have worse TNM staging (Figure 3D).

**FIGURE 4:**
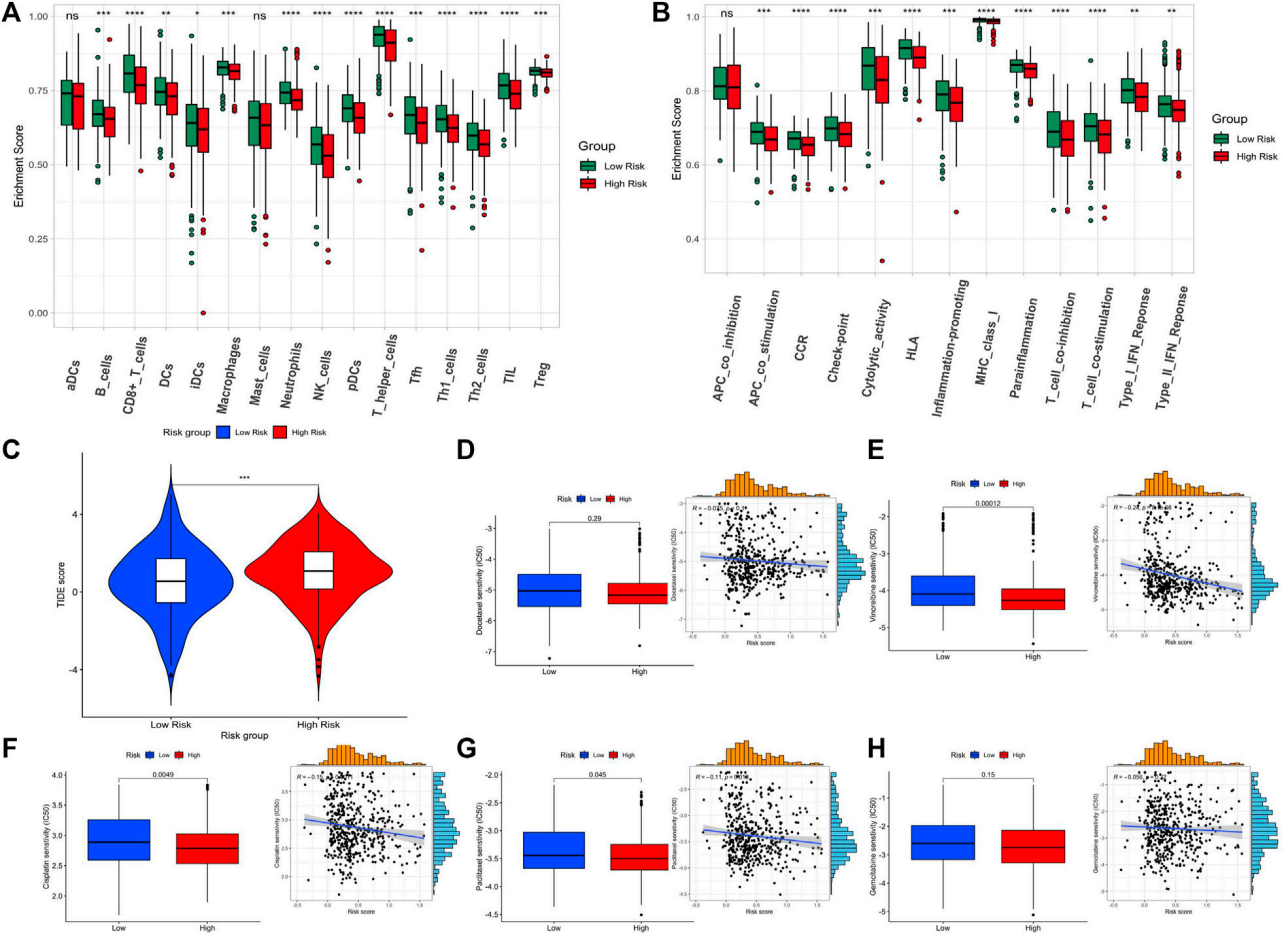
Assessment of the immune level and drug sensitivity related to the 5-lncRNA-based signature. **(A)** enrichment scores of 16 types of immune cells between the two risk subgroups. **(B)** enrichment scores of 13 immune-related pathways between the two risk subgroups. **(C)** Tide score predicting immunotherapy response between the two risk subgroups. **(D–H)** The estimated half-maximal inhibitory concentration (IC50) of docetaxel, vinorelbine, cisplatin, paclitaxel, and gemcitabine for response between the two risk subgroups.

Due to the close relationship between tumor prognosis and immunity, we further performed GSVA immune infiltration analysis with the enrichment scores of 16 types of immune cells and 13 types of immune-related pathways between the high-risk subgroup and low-risk subgroup. As the results show, the high-risk group expressed a low level of immune cells in the mass. Except for the aDCs and mast cells, significant differences appeared in the other 11 immune cells, especially in tumor-infiltrating lymphocytes (TILs), T helper (Th) cells (Th1 and Th2 cells), pDCs, natural killer (NK) cells, neutrophils, and CD8^+^ T cells (Figure 4A). In terms of the immune pathway, all of them had lower activation in the high-risk group than in the low-risk group, and only the pathway of APC co-inhibition did not show a significant difference between groups (Figure 4B). Then, we conformed to the tumor immune dysfunction and exclusion (TIDE) analysis to assess the possibility of immune escape. The result showed high-risk subgroup had a higher TIDE score than that of the low-risk subgroup, indicating that immunotherapy may be less effective in high-risk patients (Figure 4C).

Since the GSVA and TIDE analysis revealed that low-risk patients are more likely to have an active immune system and had a better prognosis after immunotherapy, we would like to assess the difference in chemotherapy response between the two subgroups. The five widely used drugs in clinical therapy for LUSC were included (docetaxel, vinorelbine, cisplatin, paclitaxel, and gemcitabine) (Figures 4D–H). We found that the estimated IC50 of cisplatin, paclitaxel, and vinorelbine chemotherapy was significantly higher in the low-risk group while that of docetaxel and gemcitabine chemotherapy had no significant difference, showing that LUSC patients in the high-risk group may be more resistant to chemotherapy of cisplatin, paclitaxel, and vinorelbine.

### Development of the Nomogram Combining the 5-lncRNA Signature and Clinical Indicators

**FIGURE 5:**
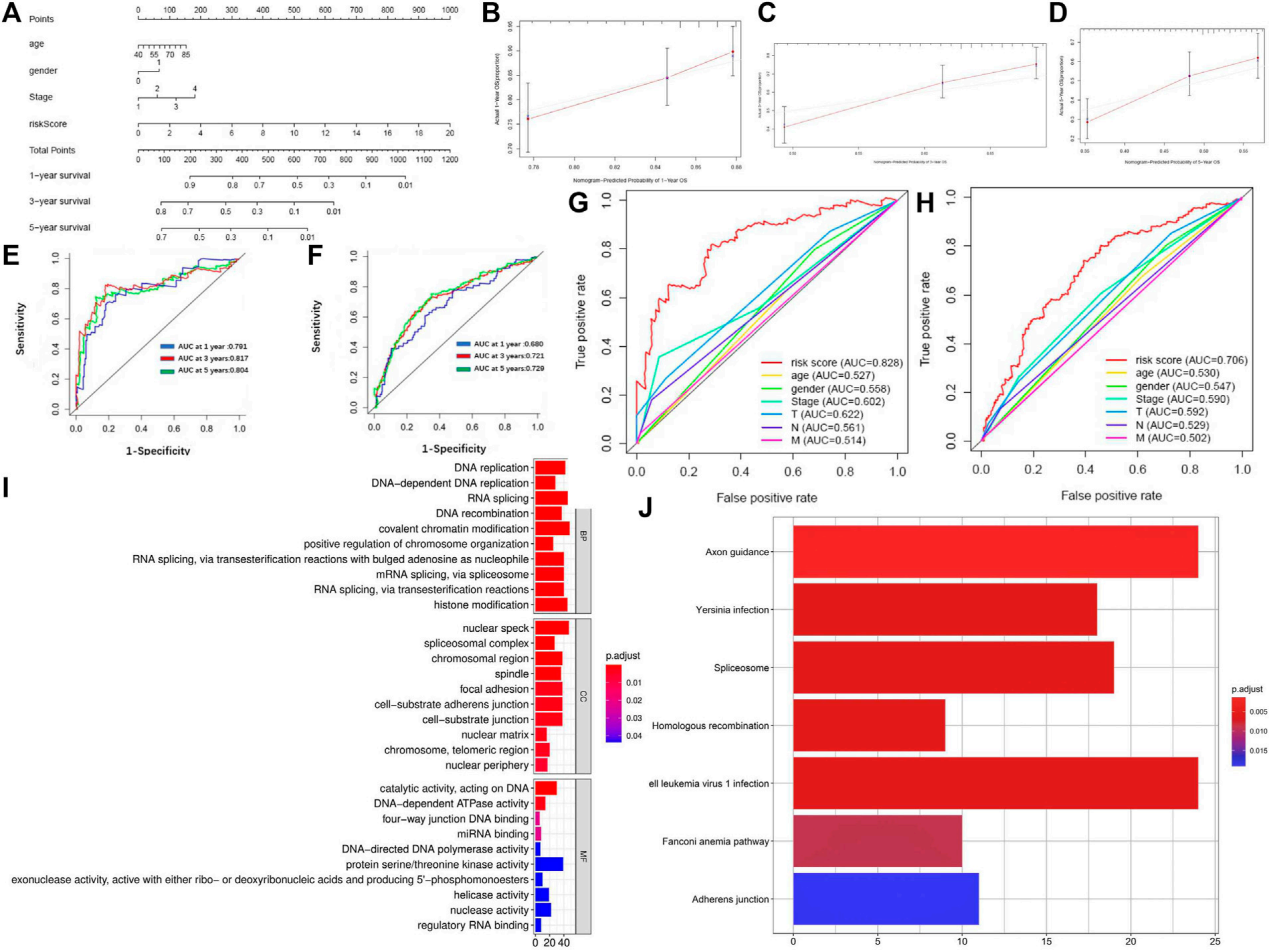
Identification of reliability and accuracy of the nomogram binding the 5-lncRNA signature and clinical indicators. **(A)** Nomogram based on 5-lncRNA in predicting prognosis of LUSC patients. **(B–D)** Calibration chart valuating the survival probability of nomogram at one, three, and five years. **(E,F)** Time-dependent ROC curves of the nomogram in the primary and entire dataset. **(G,H)** Comparison of prognostic ability between nomogram and gender, age, and TNM stage in the primary and entire dataset. **(I)** GO enrichment of mRNAs co-expressed with 5-lncRNA signature. **(J)** KEGG enrichment of mRNAs co-expressed with 5-lncRNA signature.

We drew a nomogram of the primary dataset and the entire dataset, which consisted of 5-lncRNA-based signatures and three clinical indicators (gender, age, and TNM staging) (Figure 5A). Then, we used a calibration chart to evaluate the recognition and calibration ability of the nomogram in the entire dataset (Figures 5B–D). An internal validation using bootstrap with 1000 resamplings showed that our nomogram was effective and reliable: the c-index of the primary dataset was 0.678 (95% CI = 0.613-0.743) and the c-index of the entire dataset was 0.613 (95% CI = 0.568-0.658) (Supplementary Figure S5). In addition, the AUC of the nomogram reached 0.791 at 1 year, 0.817 at 3 years, and 0.804 at 5 years in the primary dataset while 0.680 at 1 year, 0.721 at 3 years, and 0.729 at 5 years in the entire dataset, which was superior to the predictive performance of 5-lncRNA signature (Figures 5E, F). Importantly, our nomogram has a better performance (AUC = 0.828) than that of age (AUC = 0.527), gender (AUC = 0.558), TNM staging (AUC = 0.602), T staging (AUC = 0.622), N staging (AUC = 0.561), and M staging (AUC = 0.514) (Figures 5G,H).

Finally, we analyzed the co-expression mRNA of OS-related lncRNAs through GO and KEGG. 1025 DEM levels were positively correlated with the 5-lncRNA signatures (Pearson correlation coefficient >0.25). Results of GO enrichment showed that these co-expressed DEMs involved 121 biological processes, 26 cellular components, and 14 molecular functions. The mRNA functions are mainly focused on DNA replication, nuclear speck, and protein serine/threonine kinase activity (Figure 5I). Results of KEGG enrichment showed that these significantly differential co-expressed mRNAs were primarily involved in the pathway of axon guidance, Human T-cell leukemia virus 1 infection, and spliceosome (Figure 5J). GSVA enrichment analysis showed a similar result that the differential genes between high-risk and low-risk subgroups were markedly enriched in the pathway of “SPLICEOSOME”, “CELL_ADHESION_MOLECULES_CAMS” and “DNA_REPLICATION” (Supplementary Figure S6).

### SPATA41 Regulated Alternative Splicing, Apoptosis and Autophagy of LUSC Cells *In Vitro*

**FIGURE 6:**
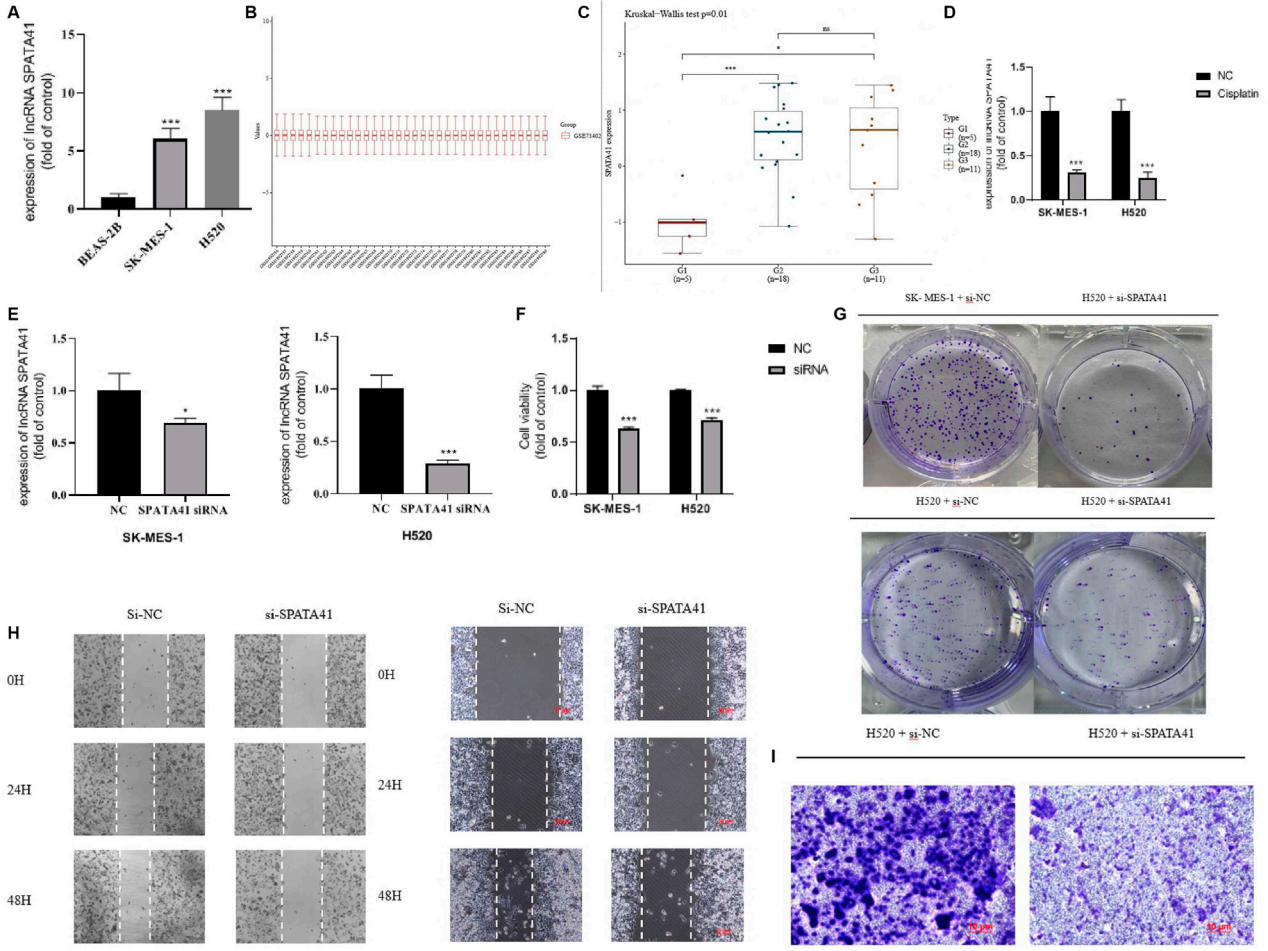
Assessment of the function of SPATA41 in cellular survival, invasion, migration, and colony. **(A)** Quantitative real-time PCR analysis of SPATA41 expression in BEAS-2B, SK-MES-1, and H520 cells. **(B)** Boxplot of the tumor, adjacent tumor, and normal lung tissue. **(C)** Comparison of SPATA41 expression in tumor, adjacent tumor, and normal lung tissue. **(D)** Quantitative real-time PCR analysis of SPATA41 expression between NC group and siRNA group in SK-MES-1 and H520 cells. **(E)** Quantitative real-time PCR analysis of SPATA41 expression between NC group and siRNA group in SK-MES-1 and H520 cells. **(F)** Results of CCK8 assays between NC group and siRNA group in SK-MES-1, and H520 cells. **(G)** Results of colony formation assays between NC group and siRNA group in H520 cells and SK-MES-1 cells. **(H)** Results of wound healing assays between NC group and siRNA group in H520 cells and SK-MES-1 cells. **(I)** Results of transwell assays between NC group and siRNA group in H520 cells. Data are expressed as mean ± SD (*n* = 3); *p < 0.05, **p < 0.01, ***p < 0.001.

We evaluated whether these OS-related lncRNAs influenced the development of LUSC. The number of DEMs co-expressed with the 5-lncRNA signature was examined, and we selected SPATA41 for further functional analysis (Supplementary Table S3). Then, two LUSC cell lines (H520 and SK-MES-1) were used to further explore the role of SPATA41 in LUSC. Quantitative real-time PCR was used to compare the expression of SPATA41 between normal lung epithelial cells BEAS-2B and that of H520 and SK-MES-1 cells. The result showed that SPATA41 expression of H520 and SK-MES-1 was higher (Figure 6A). We analyzed the differential expression of SPATA41 with LUSC related GEO database (GSE73402). Consistent with our previous result, SPATA41 expression increased significantly in tumor and adjacent tumor tissue in comparison with normal lung tissue (Figures 6B, C). We also treated H520 and SK-MES-1 cells with cisplatin, which is a special therapeutic drug for lung cancer. PCR results showed that SPATA41 was also significantly decreased by cisplatin (Figure 6D).

We then transfected SPATA41 siRNA into H520 and SK-MES-1 cells, respectively. PCR results revealed that SPATA41 expression was significantly down-regulated in H520 and SK-MES-1 cells after transfection (Figure 6E). The results of CCK8 assays also showed that SPATA41 knockdown may impair cell viability (Figure 6F). Notably, transwell assay, wound-healing assays, and colony formation assay demonstrated that the knockdown of SPATA41 dramatically attenuated the invasive, migratory, and proliferation abilities of H520 and SK-MES-1 (Figures 6G–I). We cannot get the transwell assay result of SK-MES-1 due to its low invasion ability.

**FIGURE 7:**
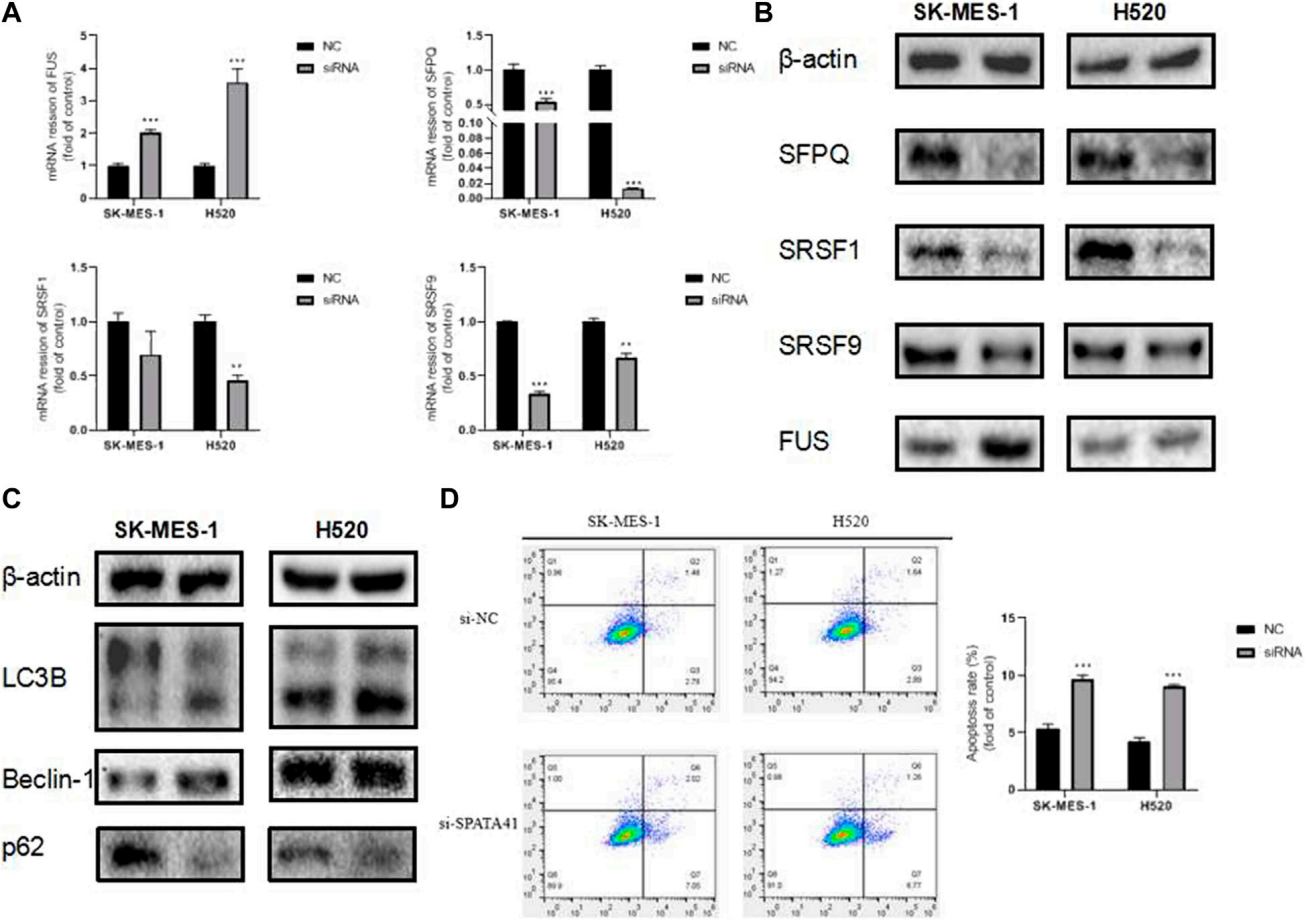
Assessment of the influence of SPATA41 on splicing factor, autophagy, and apoptosis. **(A)** Quantitative real-time PCR analysis of FUS, SFPQ, SRSF1, and SRSF9 expression between NC group and siRNA group in H520 cells, and SK-MES-1 cells. **(B)** Western blotting of FUS, SFPQ, SRSF1, and SRSF9 expression between NC group and siRNA group in H520 cells and SK-MES-1 cells. **(C)** Western blotting of LC3B, Beclin-1, and p62 expression between NC group and siRNA group in H520 cells and SK-MES-1 cells. **(D)** Results of Annexin V-FITC/PI double staining assay between NC group and siRNA group in SK-MES-1 and H520 cells. Data are expressed as mean ± SD (*n* = 3); *p < 0.05, **p < 0.01, ***p < 0.001.

Variable splicing and programmed cell death are critical processes during tumor development. To further investigate the involvement of SPATA41 in the molecular pathological course of LUSC, we predicted the possible downstream proteins of SPATA41 by CATRAPID software (Supplementary Figure S7). Finally, four potential genes (SRSF1, SRSF9, FUS, and SFPQ) were screened, which were all related to alternative splicing. PCR and WB experiments also confirmed that SPATA41 knockdown significantly affected these four splicing-associated genes (Figures 7A, B). The sequences of genes are listed in Supplementary Table S4. In addition, the expression of autophagy protein (p62, Beclin-1, and LC3B) was also influenced (Figure 7C). Results of Annexin V-FITC/PI double staining experiments also showed that knockdown of SPATA41 could cause significant apoptosis (Figure 7D). These results indicated that SPATA41 regulated the expression of splicing-associated genes in tumor cells and further influenced cellular survival.

## Discussion

Bioinformatics is a comprehensive subject of statistics, computer science, and biology (Friedlaender et al., 2019; Ren et al., 2020). It reveals the hidden biological mystery by collecting, counting, and analyzing numerous complex biological data. In the transcripts of the human genome, only 2% of the messenger RNAs encode proteins, while the rest 98% of the RNA molecules do not encode proteins, which are called noncoding RNAs (Rokavec et al., 2017). In the past, lncRNA was considered as “noise” in gene transcription. However, it has been found that lncRNA is involved in the physiological functions of cells with the development of the biological genome, including chromatin modification, post transcriptional regulation, and nuclear transport (Kourou et al., 2015).

At present, the prediction of survival time of patients with LUSC mainly depends on the TNM staging system. However, patients with similar TNM staging sometimes show opposite responses to the same treatment, which is considered caused by the heterogeneity between tumor genes (Pan et al., 2020). Therefore, an effective prognosis method for LUSC is urgently required, especially in the era of individual treatment (White et al., 2017). As people pay increasing attention to personalized medicine, many genetic markers related to LUSC prognosis have been screened (Guo et al., 2016; Ibrahim et al., 2018). However, most of these studies only focused on the statistical ability of molecular marker screening without considering its clinical effectiveness. Some studies found that in addition to classical TNM staging, gender and age are also important in predicting LUSC prognosis (Gauthier et al., 2019; Chen et al., 2020). Now in this study, we combined these clinical indicators (age, gender, and TNM staging) with 5-lncRNA signature and constructed a nomogram to quantify the survival probability of LUSC patients. We found that the predictive performance of the prognostic nomogram was better than the 5-lncRNA signature and traditional TNM staging. The c-index and calibration curve also verified the reliability of the nomogram. At the same time, simplicity is also one of its advantages, which can guide clinicians to evaluate the disease progression and prognosis more conveniently and accurately (Ferrè et al., 2016; Schmitt and Chang, 2016; Peng et al., 2017; Kopp and Mendell, 2018). Our prognostic model aimed to determine the association between prognosis and basic characteristics and should be accurate and economical (Iasonos et al., 2008; Balachandran et al., 2015; König et al., 2017; Goh et al., 2020). The nomogram included multiple independent variables and was easy for clinicians to evaluate the results and choose individual treatments for LUSC patients.

Although some famous lncRNAs have been widely reported, the specific mechanism of lncRNA still needs to be entirely explored (Li et al., 2014; Chen, 2016). The functional expression pattern of lncRNA is often related to its highly specific transcript abundance. In this study, we inferred the functions of main effective lncRNAs (SPATA41, AL034550.2, AP003721.2, AC106786.1, and AC078889.1) according to the functional evaluation of their co-expressed DEMs. Enrichment analysis of GO showed that the co-expressed DEMs were mainly enriched in DNA alternative splicing and nuclear speckles while KEGG enrichment indicated axon guidance, Human T-cell leukemia virus 1 infection, and spliceosome may be the downstream signaling pathway of the five related lncRNAs.

TME was of great importance in the initiation and development of tumorigenesis. Previous studies had identified that the immune microenvironment regulated tumorigenesis. Therefore, exploring TME remodeling has great development potential in tumor prediction and prognosis, and further fostering the transition of TME from tumor-friendly to tumor-suppressed. We found that the traditional TNM staging indicators could not distinguish the difference in TME score, while our lncRNA-based signature showed a great performance that Stromal score, Immune score, and ESTIMATE Score of low-risk subgroup were significantly higher than that of the high-risk subgroup. Our results showed that immune components in TME contributed to the prognosis of patients. Particularly, the proportion of immune and stromal components in TME was significantly correlated with the progression of LUSC, such as invasion and metastasis. These results exhibited the reliability and efficiency of our 5-lncRNA-based signature in immunity prediction.

Due to the immune-related pathways shown in functional analyses, we would like to further explore immune infiltration and immune escape between low-risk and high-risk groups. The result was consistent with our previous conclusion that the high-risk group had universally decreased levels of infiltrating immune cells, decreased activity of immune-related pathways, and higher TIDE score, indicating that low-risk patients may have opportunities for better prognosis when receiving immunotherapy. TIDE prediction scores were associated not only with poor efficacy of immune checkpoint inhibition therapy but also with poor survival of patients treated with anti-PD1 and anti-CTLA4 (Jiang et al., 2018). The lower the TIDE score was, the worse the effect of the ICB therapy may get. Importantly, we further performed an analysis of chemotherapy response to assess the drug sensitivity and resistance of our 5-lncRNA signature. The results showed that the estimated IC50 for cisplatin, paclitaxel, and vinorelbine was significantly higher in the low-risk group. High-risk patients may be more sensitive to chemotherapy of these drugs. A previous study reported that spliceosome-mediated RNA trans-splicing (SMaRT) could effectively overcome the obstacle that chemotherapy could not clear cancer cells with tumor specificity (Woess et al., 2022). Their study could explain that the different chemoresistance between groups may be related to alternative splicing.

In addition, further function assays were performed on lncRNA SPATA41, which is most associated with co-expressed DEMs among the 5 lncRNAs. Our results showed that knockdown of SPATA41 significantly affected the invasive, migratory, and proliferative abilities of LUSC cells and influenced the expression of splicing factors (SRSF1, SRSF9, FUS, and SFPQ). The apoptosis double staining experiment verified that SPATA41 knockdown could effectively induce apoptosis of SK-MES-1 and H520 cells. Moreover, increased expression of LC3 II/LC3 I and decreased expression of p62 indicated that autophagy may also be involved in the regulative process of SPATA41 in cancer cells. Thus, silencing SPATA41 in LUSC cells may prevent the development of tumors.

In conclusion, we identified the importance of lncRNA expression patterns in LUSC patients and confirmed our 5-lncRNA signature had a great advantage in assessing immune reaction, chemotherapy sensitivity, and the risk level of patients. The nomogram combining 5-lncRNA signature and clinical indicators provides an effective and reliable predictive model to help the individual treatment of LUSC patients.

## Limitation

Our study had several limitations. First, we did not refer to Lasso Cox regression to screen the differential lncRNA. Second, we did not collect clinical samples from LUSC patients for comparison. Third, our study only involved two kinds of LUSC cell lines. Fourth, we did not validate the direct interaction between lncRNA and protein by RIP or pull-down assays.

## Data Availability

The datasets presented in this study can be found in online repositories. The names of the repository/repositories and accession number(s) can be found in the article/Supplementary Material.
