# Supplementary material for: Identification of a 5-lncRNA-Based Signature for Immune Characteristics and Prognosis of Lung Squamous Cell Carcinoma and Verification of the Function of lncRNA SPATA41
Source: Front Genet. 2022 Aug 29;13:905353. doi: 10.3389/fgene.2022.905353 (PMC9465393; doi:10.3389/fgene.2022.905353)
Supplement: Supplementary file 1 [file Table1.DOCX]

| **Gene name** | **HR** | **HR.95L** | **HR.95H** | **P value** |
| --- | --- | --- | --- | --- |
| AC078889.1 | 0.502368 | 0.343729 | 0.734222 | 0.000377 |
| AL034550.2 | 0.463336 | 0.262774 | 0.816976 | 0.007848 |
| AC087521.1 | 0.00037 | 1.73E-06 | 0.078725 | 0.003863 |
| SPATA41 | 1.48881 | 1.112857 | 1.99177 | 0.007361 |
| LINC01942 | 0.024205 | 0.002079 | 0.281846 | 0.002968 |
| AP003721.2 | 0.529974 | 0.354079 | 0.79325 | 0.002032 |
| AC106786.1 | 1.641921 | 1.305936 | 2.064346 | 2.19E-05 |
| ST3GAL5-AS1 | 0.064193 | 0.010205 | 0.403784 | 0.003428 |

Supplementary Table 1. Candidate lncRNAs significantly associated with the OS of 492 LUSC patients.
