# Supplementary figures and images for: Identification of a 5-lncRNA-Based Signature for Immune Characteristics and Prognosis of Lung Squamous Cell Carcinoma and Verification of the Function of lncRNA SPATA41
Source: Front Genet. 2022 Aug 29;13:905353. doi: 10.3389/fgene.2022.905353 (PMC9465393; doi:10.3389/fgene.2022.905353)

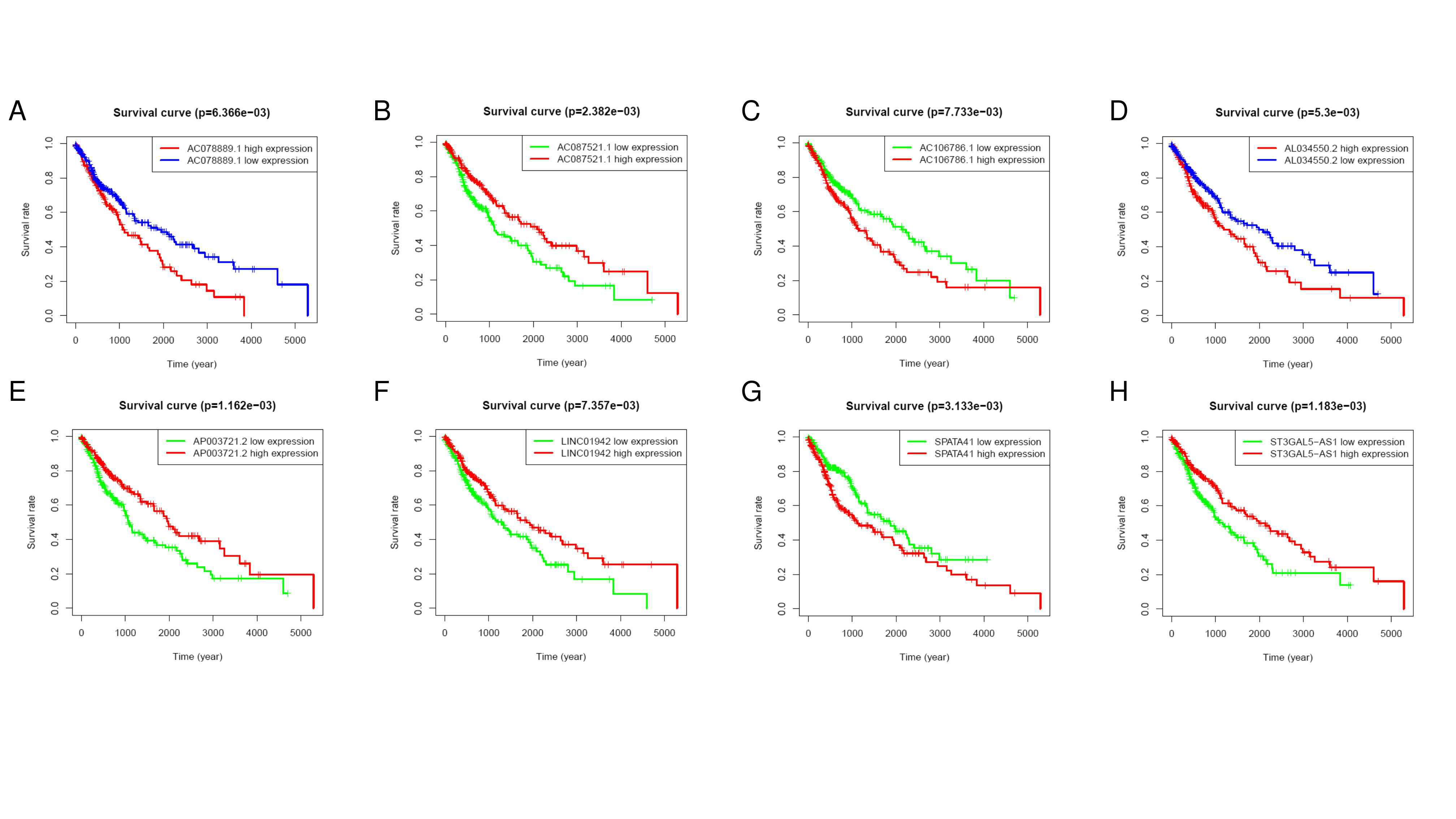

Supplement: Supplementary file 2 [file Image3.TIF]

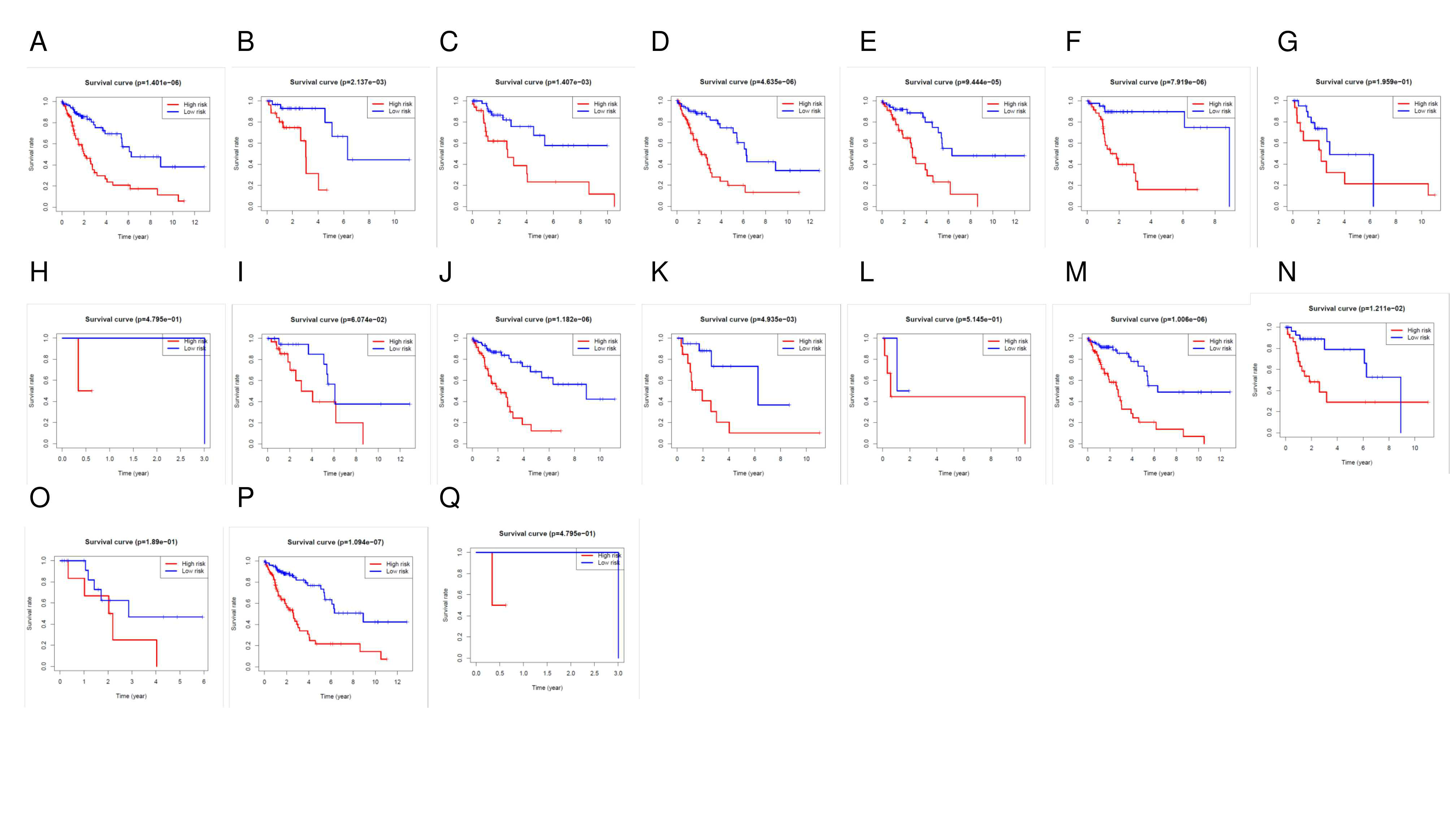

Supplement: Supplementary file 3 [file Image4.TIF]

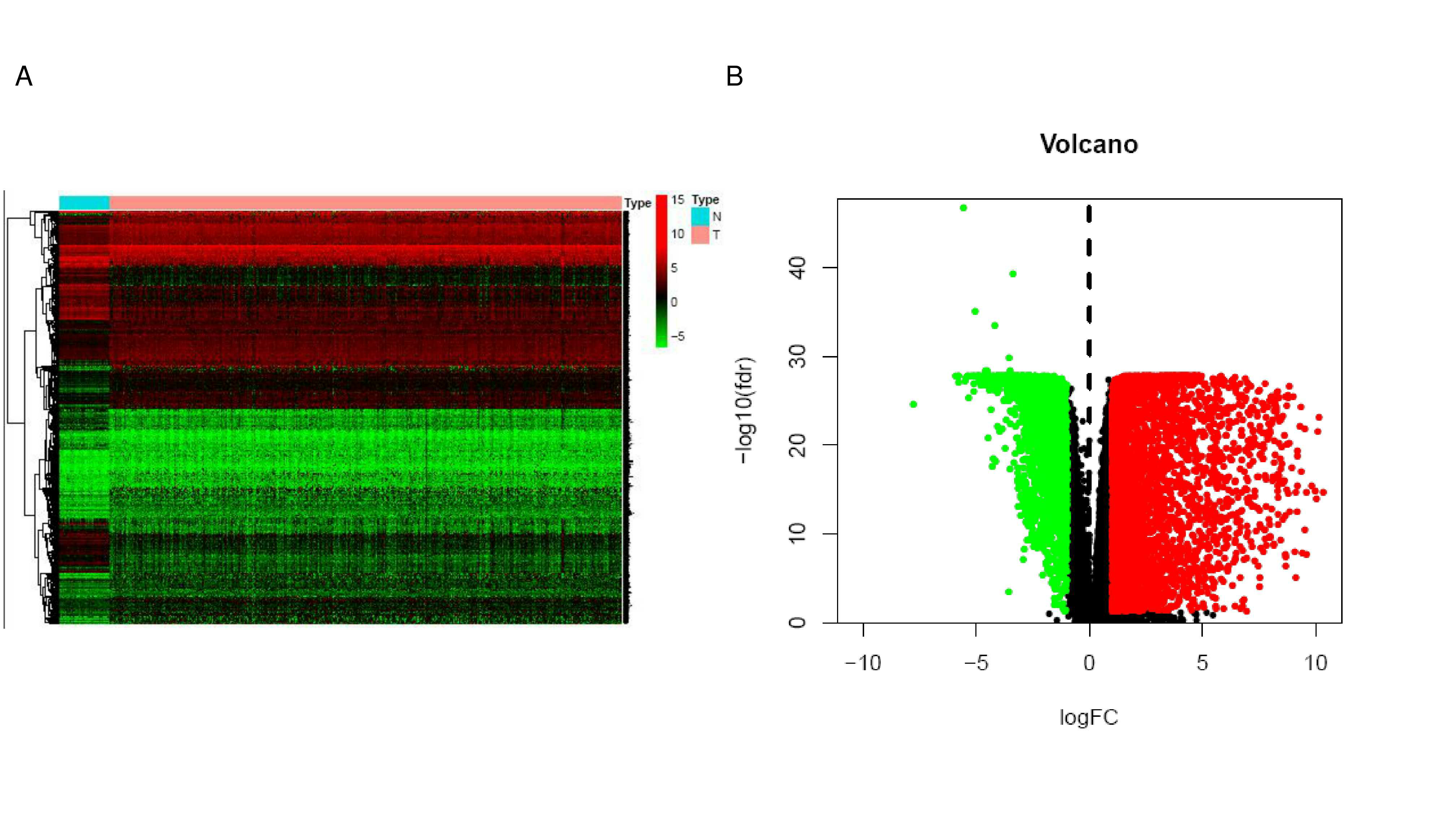

Supplement: Supplementary file 5 [file Image1.TIF]

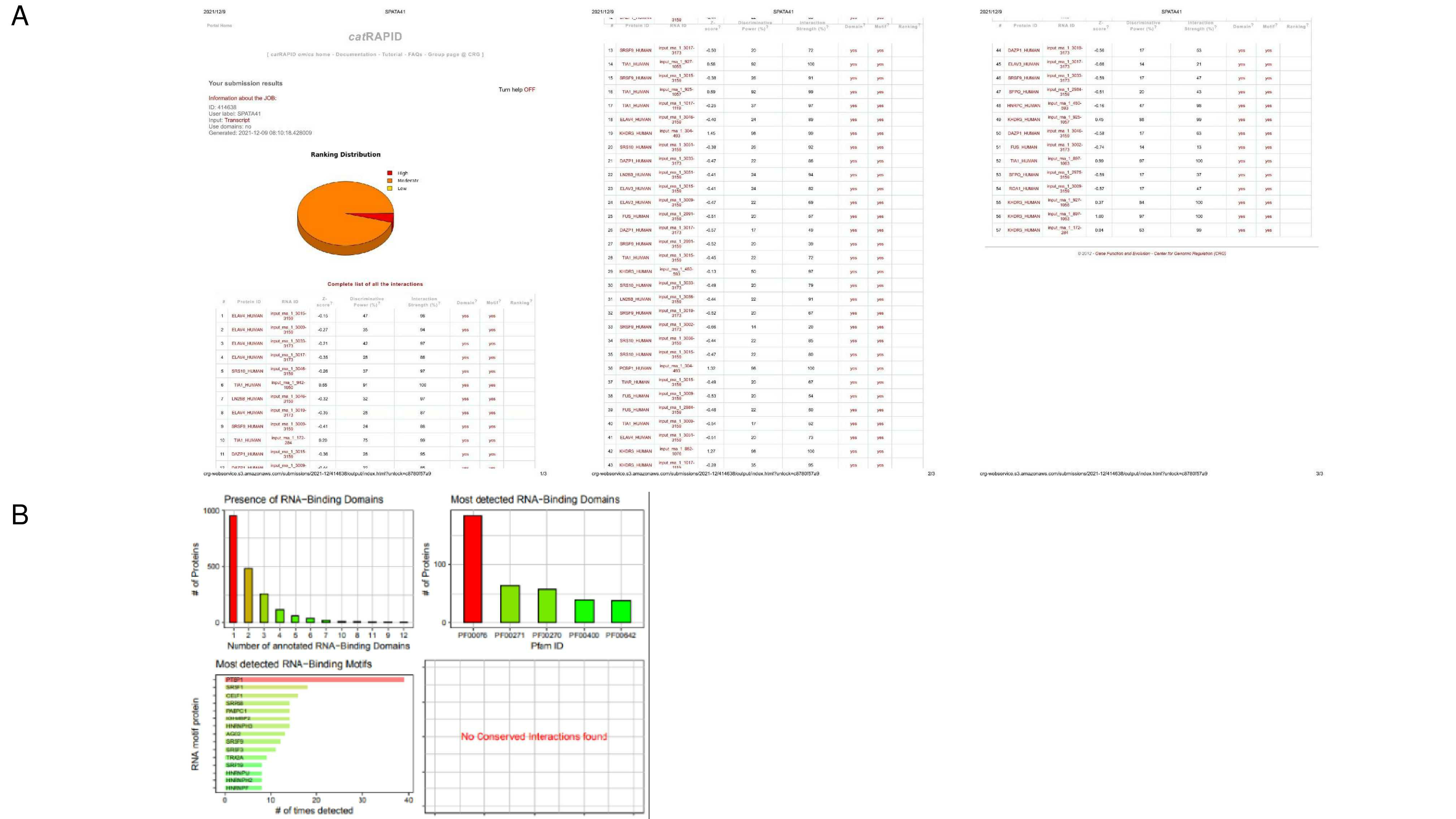

Supplement: Supplementary file 6 [file Image7.TIF]

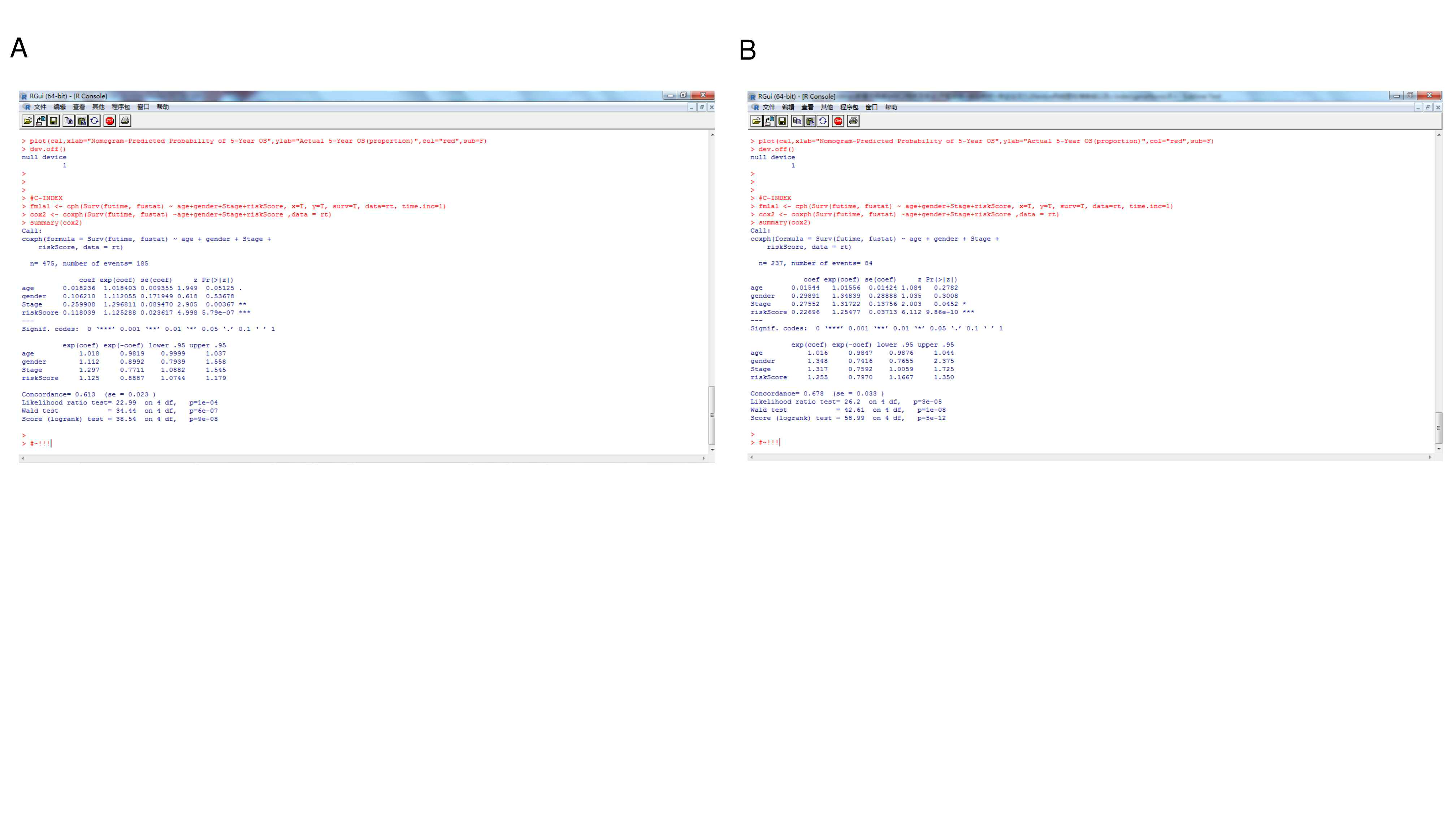

Supplement: Supplementary file 10 [file Image5.TIF]

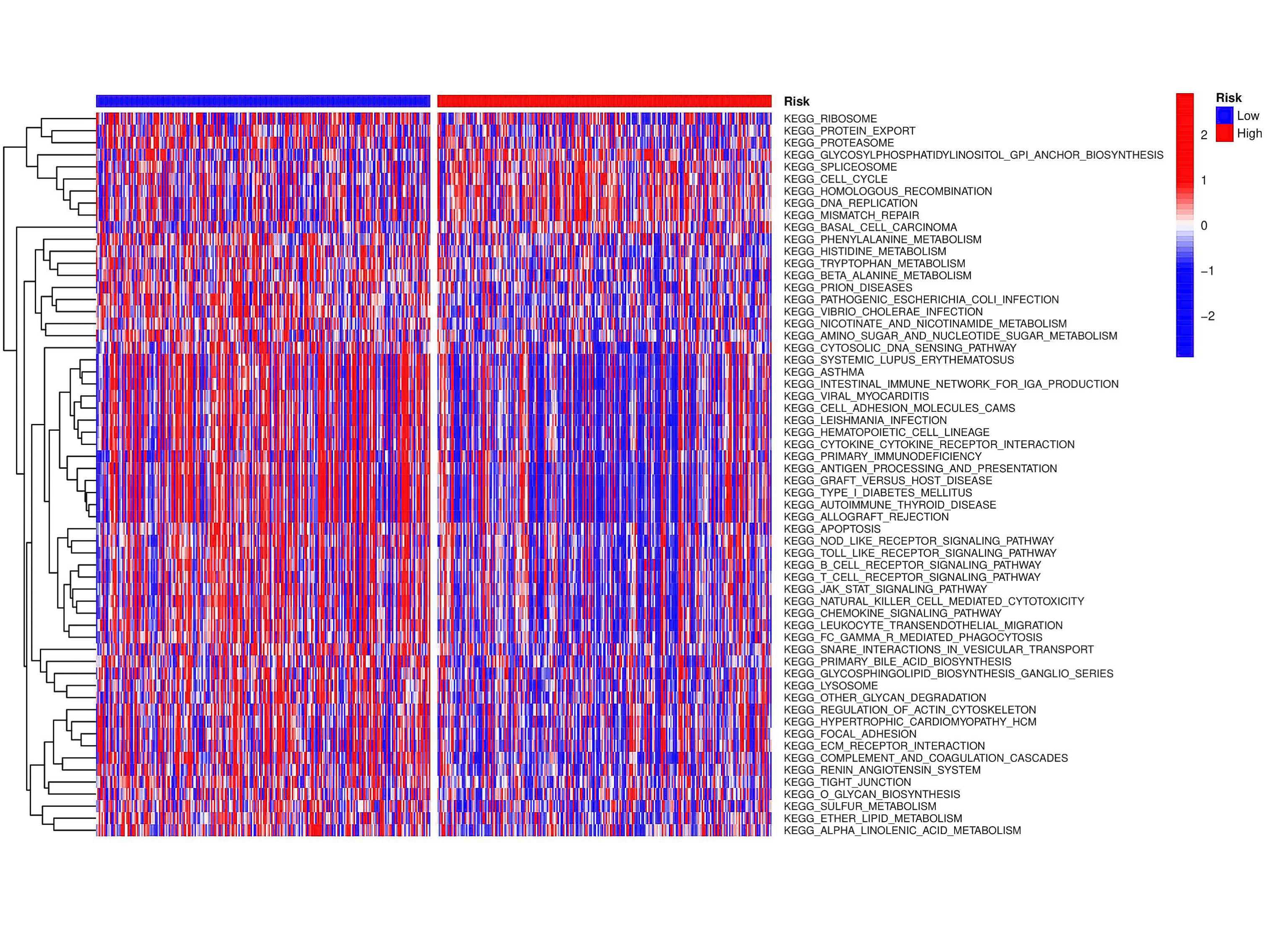

Supplement: Supplementary file 11 [file Image6.JPEG]
