# Supplementary material for: Identification of a 5-lncRNA-Based Signature for Immune Characteristics and Prognosis of Lung Squamous Cell Carcinoma and Verification of the Function of lncRNA SPATA41
Source: Front Genet. 2022 Aug 29;13:905353. doi: 10.3389/fgene.2022.905353 (PMC9465393; doi:10.3389/fgene.2022.905353)
Supplement: Supplementary file 7 [file Table2.DOCX]

| Characteristic | Univariate analysis | | Multivariate analysis | |
| --- | --- | --- | --- | --- |
|  | HR (95%CI) | P-Value | HR (95%CI) | P-Value |
| Age (≥65 vs. <65) | 1.407 (0.837-2.365) | 0.197 | 1.347 (0.791-2.295) | 0.273 |
| Gender (male *vs.* female) | 1.332 (0.760-2.334) | 0.317 | 1.237 (0.692-2.210) | 0.472 |
| TNM stage (III-IV vs. I-II) | 1.419 (1.067-1.887) | 0.016 | 1.144 (0.062-1.890) | 0.600 |
| Tumor stage (T3-T4 vs T0-T2) | 1.649 (1.170-2.324) | 0.004 | 1.508 (0.953-2.386) | 0.079 |
| Lymph node metastasis (yes *vs.* no) | 1.485 (0.709-3.110) | 0.294 | 1.643 (0.589-4.587) | 0.343 |
| Distant metastasis (yes *vs.* no) | 2.209 (0.533-9.153) | 0.275 | 1.091 (0.191-6.226) | 0.922 |
| Risk score (high *vs.* low) | 1.591 (1.391-1.820) | < 0.001 | 1.629 (1.417-1.874) | < 0.001 |

Supplementary Table 2. Univariate and multivariate CPHR analyses of the 5-lncRNA signature and clinical risk factors in the primary dataset.
