# Supplementary material for: Identification of a 5-lncRNA-Based Signature for Immune Characteristics and Prognosis of Lung Squamous Cell Carcinoma and Verification of the Function of lncRNA SPATA41
Source: Front Genet. 2022 Aug 29;13:905353. doi: 10.3389/fgene.2022.905353 (PMC9465393; doi:10.3389/fgene.2022.905353)
Supplement: Supplementary file 8 [file Table3.DOCX]

| **Gene name** | **log2FoldChange** | **Down/up-regulated** | **co-expressed mRNAs** | **P value** |
| --- | --- | --- | --- | --- |
| SPATA41 | risky | up | 966/1055  59/1025  0/1025  0/1025  0/1025 | 0.001080441 |
| AC106786.1 | risky | up |  | 0.015266432 |
| AL034550.2 | protect | down |  | 0.005590659 |
| AP003721.2 | protect | down |  | 0.004127988 |
| AC078889.1 | protect | down |  | 0.000781794 |

Supplementary Table 3. Summary of the five OS-related lncRNAs.
