# Supplementary material for: Identification of a 5-lncRNA-Based Signature for Immune Characteristics and Prognosis of Lung Squamous Cell Carcinoma and Verification of the Function of lncRNA SPATA41
Source: Front Genet. 2022 Aug 29;13:905353. doi: 10.3389/fgene.2022.905353 (PMC9465393; doi:10.3389/fgene.2022.905353)
Supplement: Supplementary file 9 [file Table4.DOCX]

| **Gene name** | **The sequences of mRNA** |
| --- | --- |
| SRSF1 | \| Sense: ATGGAAGATCTCGATCTCGAAGC Antisense: TTATGTACGAGAGCGAGATCTGC \| \| --- \| |
| SRSF9 | \| Sense: GGATGTCTGTTATGCTGATGTGC Antisense: ACTCGGATGTAGGAAGTTTCACC \| \| --- \| |
| SFPQ | \| Sense: GAGATGGAAGAACAAATGAGGCG Antisense: ATTTCTGGCCTCCTGAACCATAG \| \| --- \| |
| FUS | \| Sense: GGACAGCAGAGTTACAGTGGTTA Antisense: CTGAGTTCCATAGCCTGTGTTCT \| \| --- \| |

Supplementary Table 4. Sequences of PCR primers.
